# Influence of Infill Level and Post-Processing on Physical Parameters and Betaine Content of Enriched 3D-Printed Sweet Snacks

**DOI:** 10.3390/foods12244417

**Published:** 2023-12-09

**Authors:** Kristina Radoš, Kristian Pastor, Jovana Kojić, Saša Drakula, Filip Dujmić, Dubravka Novotni, Nikolina Čukelj Mustač

**Affiliations:** 1 Faculty of Food Technology and Biotechnology, University of Zagreb, Pierottijeva 6, 10000 Zagreb, Croatia; kristina.rados@pbf.unizg.hr (K.R.); sasa.drakula@pbf.unizg.hr (S.D.); filip.dujmic@pbf.unizg.hr (F.D.); nikolina.cukelj@pbf.unizg.hr (N.Č.M.); 2 Faculty of Technology Novi Sad, University of Novi Sad, Bul. Cara Lazara 1, 21000 Novi Sad, Serbia; 3 Institute of Food Technology in Novi Sad (FINS), University of Novi Sad, Bul. cara Lazara 1, 21000 Novi Sad, Serbia; jovana.kojic@fins.uns.ac.rs

**Keywords:** snack products, food 3D-printing, gluten-free, texture, sensory analysis, porosity, dimensions

## Abstract

Betaine is a non-essential amino acid with proven functional properties and untapped potential for cereal food enrichment. While 3D printing represents a viable approach for manufacturing enriched cereal-based foods with novel shapes and textures, it is crucial to consider the impact of printing parameters and post-processing on the betaine content and properties of these products. The aim of this study was to investigate the influence of the infill level (20, 30 and 40%) of 3D-printed cuboid shapes and the post-processing techniques (drying oven, vacuum dryer, air fryer) of betaine-enriched oat-based snacks on the print quality, texture, and sensory properties, as well as the content of preserved betaine. The interaction of post-processing technique and infill level influenced the length deviation and texture properties, as well as the betaine content of snacks. Height stability was only influenced by post-processing technique. In general, oven-dried snacks showed the best dimensional stability, having the lowest width/length deformation (about 8%) at the infill level of 20%. Betaine was best preserved (19–31% loss) in snacks post-processed in a vacuum dryer (1281–1497 mg/g), followed by an air fryer and a drying oven, where betaine loss was in the range 28–55%. Air-fried snacks with 40% infill level had the highest values of instrumentally measured crunchiness (38.9 Nmm) as well as sensory test values for liking of texture (7.5), intensity of odor (6) and overall flavor (6). Overall, air frying proved to be a convenient and quick post-processing technique for 3D-printed snacks, but infill patterns for preserving betaine should be further explored. Vacuum drying could be used to preserve bioactive compounds, but efforts should be made to minimize its negative impact on the physical deformations of the 3D-printed products.

## 1. Introduction

The content and composition of our daily diet has a notable influence on various bodily functions and can either promote or mitigate certain diseases [1]. This awareness has sparked an expanding fascination with enriched and fortified foods worldwide, propelled by rising healthcare costs and increasing human life expectancy. Snacks are usually enjoyed between meals to satisfy hunger or fulfill cravings. They come in various forms and tastes and are often designed to be convenient on the go and, thus, packed in small portions for individual consumption. The global market for healthy enriched snacks is expected to reach about 33 billion US dollars by 2025 [2]. Snack products are designed to prioritize quick energy sources [3], and those that are cereal-based additionally serve as excellent candidates for enrichment and fortification [4]. Betaine is a compound naturally found in foods, such as whole grains, beets and spinach, with a range of health benefits. These include supporting heart health, bolstering liver function, improving physical performance, improving cognitive function in older adults and reducing inflammation in the body [5]. Betaine is usually extracted from sugar beet or sugar cane molasses [6,7]. It is a generally recognized as a safe (GRAS) ingredient in the United States and it has been approved by the European Food Safety Authority for use in food at a maximum level of 6 mg/kg body weight per day [8]. The inclusion of betaine in snacks could deliver this compound to a wider population, increase its use as part of a balanced diet, and potentially support overall health and well-being [5]. Although supplementation of betaine through various enriched foods appears promising, the role of processing on betaine stability should not be neglected.

Three-dimensional (3D) printing technology has attracted significant interest from researchers and the food industry, particularly in the development of customized snack products [4]. There are several 3D printing technologies that can be used in the food industry, including selective sintering technology, binder jetting, ink jet printing as well as hot melt extrusion, which is the most commonly used and is suitable for many food materials [9]. It offers unprecedented design flexibility and precise control over product geometry, opening up new possibilities for customization and personalized nutrition including food enrichment [9,10,11,12,13,14]. For example, 3D-printed cereal snacks enriched with button mushrooms [15], microalgae [16,17], wheat bran [18], malt and whole-grain flour [19] have been proposed.

Cereal products generally require thermal processing, such as cooking, drying, baking, roasting, or toasting to improve their digestibility, storage stability and sensory properties. On the other hand, thermal processing can lead to the degradation of biological active compounds, including betaine [20]. It is, therefore, important to accurately determine the betaine content in the final product. This will ensure that the amount of betaine indicated on the label is reliable, allowing an appropriate portion recommendation and an assessment of the potential health benefits. Moreover, the appearance, texture, and flavor of 3D-printed snacks can be greatly affected by post-processing temperatures [21,22]. Although there are a few studies using different infill levels and patterns to modify texture of 3D-printed snack products [23,24,25], none of them investigated how different post-processing techniques and infill levels influence the preservation of bioactive compounds, such as betaine.

Thermal processing of cereal-based snacks generally involves intricate heat transfer phenomena that affect the products’ internal and surface temperature profiles, melt flow behaviors and crystallization patterns [26]. Although efforts have been made [14,22,27,28], the effects of thermal steps on the microstructure and physicochemical attributes of 3D-printed cereal-based snacks remain poorly characterized, especially in relation to different geometric structures. Previous studies have mainly focused on the dimensional stability of 3D-printed products, while texture, sensory acceptability, and especially the preservation of heat-sensitive bioactive compounds have not been studied [23]. Although drying is still the most commonly used post-processing method of 3D-printed snacks [29], there is a crucial need for a broader understanding of alternative post-processing methods.

The aim of this study was, therefore, to investigate the influence of three different infill levels and three different post-processing methods (air frying, oven drying, vacuum drying) on the quality of the betaine-enriched 3D-printed oat-based snack. Physical properties, such as geometric accuracy, shape stability, porosity and texture (firmness, crunchiness) were investigated together with the betaine content. Finally, to assess the impact of the infill level and post-processing of the snacks, sensory evaluation was carried out.

## 2. Materials and Methods

### 2.1. Materials

Oat flour (9.5% protein, 5.6% lipids, 2.2% fiber) (Garden Ltd., Zagreb, Croatia), defatted hazelnut flour (41.6% protein, 17% lipids, 16.7% fiber) (Biovega Ltd., Zagreb, Croatia), rice protein (80.1% protein, 1.8% fats, 4.3% fiber) (BioFUN Ltd., Sarajevo, Bosnia and Herzegovina), powdered sugar (Ultragros Ltd., Zagreb, Croatia), cinnamon powder (Podravka Inc., Koprivnica, Croatia), psyllium husk powder (Biovega Ltd., Zagreb, Croatia), Omegol sunflower oil (Zvijezda plus Ltd., Zagreb, Croatia) and tap water were used for preparing snacks. Anhydrous betaine was used as a supplement (98%, AlfaAesar GmbH&KG, Karlsruhe, Germany).

### 2.2. Methods

#### 2.2.1. Dough Preparation

Firstly, oat (30 g) and hazelnut (11.5 g) flour were mixed with rice protein (6 g), sugar (5 g) and cinnamon (0.5 g). Betaine (1.7 g) was dissolved in tap water (30 mL) using vortex, and psyllium powder (0.9 g) was added. The obtained gel was mixed with the dry ingredients together with oil (6 g) for 3 min using a hand mixer (M350 LBW, Gorenje, Slovenia) to prepare the dough.

#### 2.2.2. Dough Rheology Measurements

Oscillatory measurements were undertaken in two replicates using an MCR 92 rheometer (Anton Paar, Graz, Austria) with a parallel plate measuring device (PP25) at a 1 mm gap. Frequency and amplitude sweep tests were performed as previously described by Habuš et al. [18]. After measurements, the storage modulus (G′), loss modulus (G′′), loss factor and complex viscosity were recorded. A three interval thixotropy test (3ITT) was also performed to determine the effect of instant interfacial shear rate on deformation and regeneration of the dough after printing. The deformation kinetics of the dough were examined in three steps. In the first step, no shear was applied (resting dough), while in the second step, shear stress was applied (simulation of the printing process), and again in the third step, no stress was applied (dough after printing). In the second step, the sample was deformed with a shear rate value of 15/s for 20 s (printing of one layer takes around 20 s). The deformation (in percentage) was calculated using the following equation:

% Deformation (% Dr) = Gi − G0/Gi × 100

where Gi represents the initial G′ value of the samples and G0 indicates the G′ values immediately after shear strain deformation [30].

#### 2.2.3. Experimental Plan, 3D Printing and Post-Processing

Snacks were prepared following a full-factorial experimental design, using three infill levels and three post-processing methods (Table 1). Printing of 50 pieces (5 syringes of dough) of each sample was performed on the extrusion-based Foodbot D2 Multi Ingredient Dual Head Food 3D Printer (Changxing Shiyin Technology Co., Ltd., Hangzhou, China) at a print speed of 10 mm/s and the temperature set at 30 °C, using 0.84 mm nozzle. Computer-aided design (CAD) models were designed using a 3D CAD design software Solidworks (Dassault Systèmes, France) and sliced to G-code using Slic3r (open-source software). The dimensions of selected cuboid shape were 1.7 × 1.7 × 0.7 cm for width, length and height, respectively, which resulted in a shape consisting of seven layers. Post-processing of the printed samples was performed using a drying oven with gravity convection, without air flow, at 130 °C (HERATHERM, Thermo Fisher Scientific, Waltham, MA, USA), an air fryer, which is combination of radiation and convection with air flow at ~180 °C (ACTIFRY FZ706039, Tefal, France), and a vacuum dryer at 80 °C and 100 mbar (VO 200, Memmert, Germany). In preliminary experiments, the post-processing time was fixed (Table 1) to achieve a moisture content below 5% [22], which was determined using a PMB moisture analyzer (Adam Equipment, Milton Keynes, UK), i.e., the sample was dried until a stable weight was reached.

Fifteen sample pieces were post-processed simultaneously and used for further analysis. The output variables were mass, moisture content, width, length, height, texture (firmness, crunchiness, brittleness), porosity and betaine content. Samples for the sensory analysis were printed in four batches, packed in laminated aluminum bags and served to panelists the day after post-processing.

#### 2.2.4. Moisture Content Determination

The moisture content of the post-processed 3D snacks was determined at 130 °C for 3 h (Thermo Scientific, Heratherm), as described by Agarwal et al. [31]. Measurements were performed in two replicates.

#### 2.2.5. Betaine Determination

The quantification of betaine content was carried out following an optimized and validated method using an HPLC system (Agilent Technologies Inc., Santa Clara, CA, USA) equipped with a Kinetex HILIC (Phenomenex, Aschaffenburg, Germany) column (2.6 mm, 100 × 2.1 mm) and ELSD detector (1290 Infinity ELSD, Agilent Technologies Inc., Santa Clara, CA, USA). The analysis was performed with a flow rate of 0.5 mL/min, using an 80:20 mixture of acetonitrile and 10 mM acetate buffer (pH 3.7) as the mobile phase. The injection volume was 5 µL, and the total run time was 10 min. The method was developed, optimized, and validated for cereals and pseudocereals as comprehensively elaborated by Kojić et al. [20]. All the samples were analyzed in two replicates. The loss of betaine was determined from the difference between the calculated expected betaine content in the 3D-printed snack and the analytically measured values.

#### 2.2.6. Texture Analysis

The texture (firmness, brittleness and crunchiness) of the post-processed 3D-printed snacks was analyzed using a texture analyzer (TA1, Ametek Lloyd Instruments Ltd., Bognor Regis, UK) equipped with a 50 kg load cell and the Warner–Bratzler shear blade guillotine probe. The probe was positioned a few mm above the sample. The test was performed with a preload stress of 0.245 N and test speed of 2 mm/s [18] in ten replicates at least four hours after post-processing. It was specified in the settings to stop the test when sample breaks or at 90% of shear. The texture parameters were calculated with the Nexygen 4.0 software, which is connected to the texture analyzer.

#### 2.2.7. Sensory Analysis

The sensory analysis of the snacks was carried out at the Faculty of Food Technology and Biotechnology at the University of Zagreb, Croatia, according to ISO 6658:2017 and ISO 13299:2003 (Sensory Analysis—Methodology—General Guidance, International Organization for Standardization: Geneva, Switzerland), with employees as assessors. The analysis was conducted in accordance with the Declaration of Helsinki, and the protocol was approved by the Ethics Committee of the University of Zagreb Faculty of Food Technology and Biotechnology. The analysis consisted of two separate sessions—evaluation of infill level followed by evaluation of post-processing technique. Hedonic and ranking tests were used to select the most favourable infill level. This evaluation included 12 assessors with snacks post-processed in a drying oven. In the next session, the 3D-printed snacks with the preferred infill level were post-processed using three different techniques (as described in Section 2.2.4.). These snacks were evaluated using descriptive analysis, hedonic and ranking tests by 16 assessors (aged from 36 to 60 years). Immediately before the session, the panelists were trained using a sample with previously defined intensities. The panelists were then offered two pieces of each sample, coded using three-digit numbers, and served with water to rinse the palate. First, they evaluated appearance (color intensity) and odor, then ingested the sample and evaluated the flavor and texture characteristics.

In the hedonic test, the snacks were evaluated according to appearance, texture and overall perception on a scale from 1 (extremely dislike) to 9 (extremely like). In the ranking test, the assessors ranked the snacks from the most preferred (score 1) to the least preferred (score 3). In the descriptive test, assessors evaluated the intensity of sensory attributes of the snacks on a scale from 0 (not perceived) to 10 (very intense). The evaluated attributes included appearance (color intensity), odor (overall), flavor (overall, hazelnut, bitter taste), texture (hardness—the force required to bite the product with the incisors; and fracturability—the force required to break the product into crumbs or pieces by forced pressure between the incisors).

#### 2.2.8. Image Analysis

**Figure 1:**
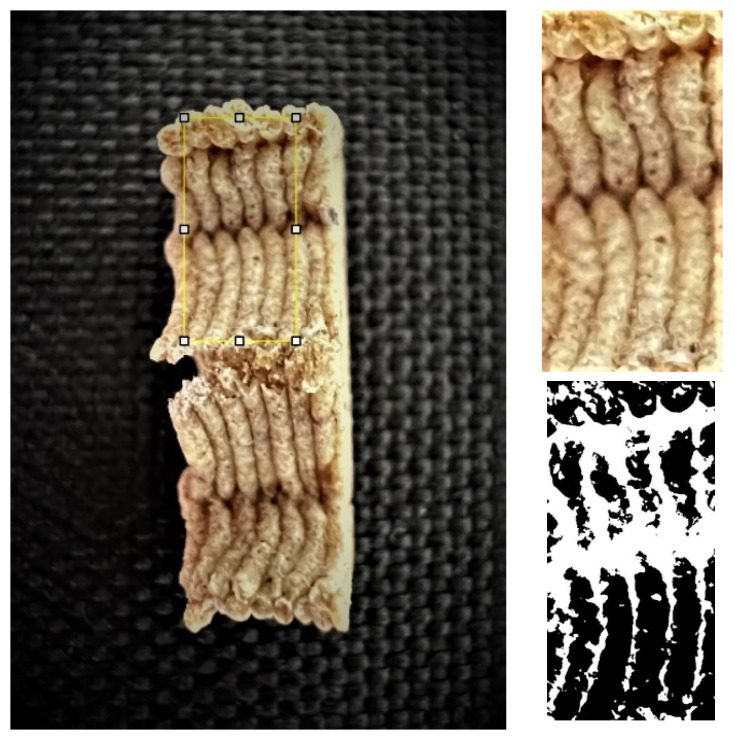
Porosity assessment of an example snack (printed with 20% infill density and post-processed in an air fryer): selection on a cross-section of the whole sample (**left**); cropped and magnified selected part (**upper right**); and pore area assessment (white area) on a binary 8-bit picture (**below right**).

Digital images of the 3D-printed snacks were taken to obtain side, above and cross-sectional views using a smartphone (Iphone 13 Pro, Apple, SAD) at a fixed distance of 13 cm under daylight. The device consisted of a triple camera system with the following specifications: 12 MP sensor-shift OIS, aperture size F1.5, focal length 26 mm, sensor size 1/1.65”, pixel size 1.9 µm (first camera); 12 MP telephoto OIS, optical zoom 3.0x, aperture size F2.8, focal length 77 mm (second camera); and 12 MP ultra-wide autofocus, aperture size F1.8, focal length 13 mm (third camera). An open-source image processing and analysis software in Java, ImageJ v.1.53e (National Institutes of Health, NIH, USA), was employed to measure width (horizontal side), length (vertical side), and height (cm) and calculate height stability, width and length deviation (%). Measurements were performed in five replicates. The dimensional deviation of the printed dough was compared with the design dimensions. The dimensional deviation of post-processed samples was calculated against the dimensions of the printed dough. Image J was employed to assess the porosity of the cross-sectional surfaces of the 3D-printed snack samples, as depicted in Figure 1. The initial images underwent cropping to isolate the porous region within the sample cross-section. Subsequently, they were converted to an 8-bit grayscale format to improve contrast. The threshold value was individually determined for each sample, beginning from zero and incrementally increasing until reaching the initial peak in the threshold graph. Material porosity was then quantified as the proportion of the white area relative to the total sample surface. Measurements were performed in five replicates. The details about the calculation process of 3D-printed snack dimensional properties can be found in Čukelj Mustač et al. [32].

#### 2.2.9. Data Analysis

The influence of infill level and post-processing technique on the output values was analyzed using analysis of variance (ANOVA) followed by a Tukey post hoc test at the 0.05 level of probability. In addition, a Pareto chart of standardized values was used to evaluate if infill level or post-processing had a greater effect. The experimental design, ANOVA, Tukey test, Spearman correlation test and Pareto charts were performed using the software system Statistica v. 13 (Tibco Statistica, Palo Alto, CA, USA).

## 3. Results and Discussion

### 3.1. Physical Properties and Printing Quality of Dough

The rheological analysis of the dough confirmed its viscoelastic behavior. The elastic modulus (G′, Pa), viscous modulus (G″, Pa), loss factor (tanδ = G″/G′), and complex viscosity (η∗, Pas) at a frequency of 1 Hz were 102425 ± 3195 Pa, 34922 ± 1326 Pa, 0.314 ± 0.002, and 7101 ± 210 Pas, respectively. The percentage deformation of the dough after the application of shear stress was 23%. Values of loss factor less than 1 indicate a predominantly elastic behavior [16]. A similar ratio of G″/G′, where G′ is 3–4 times higher than G″, and comparable values of η∗ (7970 Pas) were also found in a previous study on a similar type of dough [31]. These values ensure that the printed dough layer can support the weight of the next layer [25,33], which is crucial for the success of printing. Thus, the dough was smoothly extruded during printing and exhibited good structural stability. However, an oozing phenomenon of the dough filament was also visually observed. Oozing primarily occurred due to the non-printing movement of the nozzle during the printing process. In extrusion printing of thermoplastic materials, this defect can be remedied by actively retracting the filament between extrusions, that is, before non-printing movement. However, this does not apply to food printing. Namely, commonly used slicing software Slic3r is mainly adapted and optimized for the physical properties of thermoplastic materials, regardless of food material [34].

The dimensions and shapes of the printed dough as well as the designed CAD models are shown in Table 2. As expected, shape weight increased with higher infill levels: samples with 40% infill level were around 30% heavier than those with 20% infill, which weighed 0.9 g on average. This comes as no surprise since a higher infill percentage implies deposition of more material for building the internal structure of the product [23]. The printed shapes matched the designed shapes with some deviations in dimensions. The infill level had a significant (*p* = 0.004) influence on width, length and height of the samples. Compared with the CAD model, the length/width dimensions were slightly smaller (1–2%) in samples with 20% infill, while they were 7% larger in samples with 30% and 40% infill levels. This probably came from the higher number of dough filaments creating 30 and 40% infill, which pushed the shell of the object and contributed to the higher deviations. The height stability of samples with 20% infill was the lowest (on average 87%), while samples with 30% infill were the most stable (on average 91%). Liu et al. [34] found that varying the infill level from 10 to 40 and 70% had no significant effect on the height of snacks made of potato flakes and wheat flour, but increasing the infill from 10 to 40% significantly influenced the diameter of the snacks. In contrast, Huang et al. [29] used a wider range of infills (15, 45 and 75%) of brown rice snack dough to establish that the infill density had little or no effect on the dimensional properties, but only changed what was printed inside the shell of the objects. The influence of infill level on the dimensional stability may, therefore, be only visible at lower infill levels (up to 40%), although the dough rheological properties should also be taken into account.

### 3.2. Physico-Chemical Properties of Post-Processed Snacks

**Figure 2:**
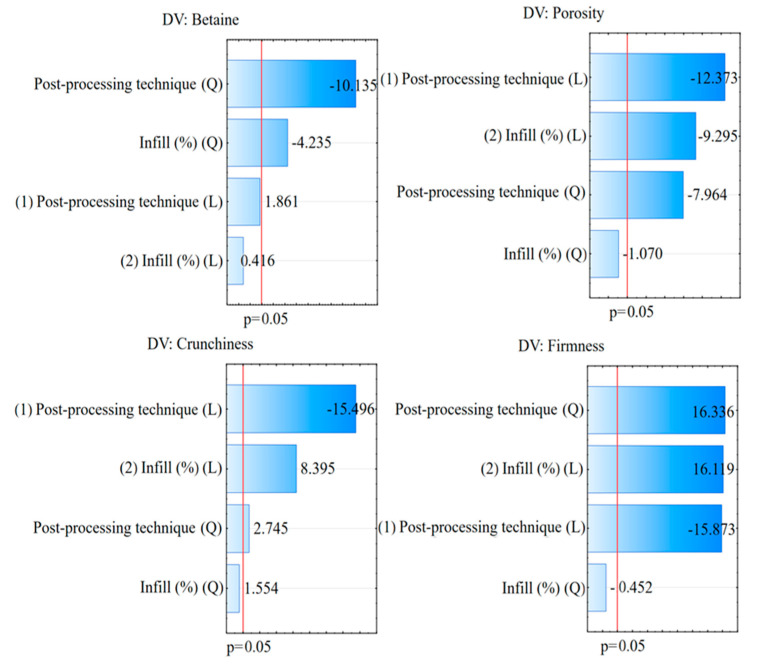
Pareto charts for the estimation of independent variables (post-processing technique and infill) on: betaine, porosity, crunchiness, and firmness. Higher values of standardized effect estimates represent a more pronounced effect on the response. Red lines represent the *p* = 0.05 probability level; all values situated on the right of the red lines represent significant effects, while the values on the left represent effects with no statistical significance. Letters L and Q in the brackets represent the estimation of the linear (L) and the quadratic (Q) effect.

The analysis of variance (ANOVA) showed that, in general, both the infill level and post-processing technique had a significant influence on the investigated physico-chemical parameters of the 3D-printed snacks: moisture and betaine content/loss, printed shape dimensional stability, porosity, and texture (Table 3). Moreover, the Pareto charts show that post-processing technique had a greater effect than infill level on betaine content, porosity, crunchiness and firmness (Figure 2).

AF-20—snack with 20% infill level, post-processed in air fryer; AF-30—snack with 30% infill level, post-processed in air fryer; AF-40—snack with 40% infill level, post-processed in air fryer; DO-20—snack with 20% infill level, post-processed in drying oven; DO-30—snack with 30% infill level, post-processed in drying oven; DO-40—snack with 40% infill level, post-processed in drying oven; VD-20—snack with 20% infill level, post-processed in vacuum dryer; VD-30—snack with 30% infill level, post-processed in vacuum dryer; VD40—snack with 40% infill level, post-processed in vacuum dryer.

#### 3.2.1. Moisture and Betaine Content of Snacks

The moisture content of the snacks was below 5%, as recommended for cereal-based snacks [35], and ranged from 2.2% for DO-40 to 4.5% for VD-20 (Table 3). Since the method used only tracked the weight of the samples at the same time intervals until it was unchanged, but not the exact moisture content throughout the post-processing period, a wider range of moisture content was obtained in the final products. A narrower time interval and a constant determination of moisture content should be considered in the future. To achieve a constant weight of product, the AF snack required significantly less time, compared with the DO and VD snacks, which needed a maximum of 30 and 90 min, respectively (Table 1). 

Although betaine is known to withstand harsh treatment during sugar beet processing, cooking and baking may result in loss of betaine in enriched foods, although it is thermostable in pure form [36]. In this study, the betaine content ranged from 845 to 1497 mg/100 g for DO-40 and VD-40, respectively. It was influenced by the interaction of infill level and post-processing technique (*p* < 0.001), where the post-processing technique exhibited a dominant quadratic effect (Figure 2. With increasing infill level, betaine loss was reduced in the VD and AF snacks, while it was increased in the DO snacks. Thus, the highest loss of betaine (55%) occurred in the snack with and infill level of 40% post-processed for 30 min in DO at 130 °C. On the other hand, the highest amounts of betaine were preserved when the 3D-printed snacks were post-processed in the VD at 80 °C for 90 min. As this was the longest post-processing time used in this study, the importance of post-processing temperature in addition to drying time becomes clear. Indeed, AF, which operated at the highest temperature but for the shortest time, resulted in a higher betaine loss compared with VD (Table 3). It is also interesting to notice that the betaine loss in AF was lower with higher infill levels. This could be due to the fact that AF produces extremely high heat transfer rates during which the hot air is also distributed uniformly through the product at high velocities [36], resulting in lower losses of betaine for snacks with denser structure and less open surface. Previous studies reported varying levels of betaine losses during thermal processing. For example, during the baking of scones, de Zwart et al. [37] observed a 17% reduction in betaine content, and similar losses were observed in betaine-fortified gluten-free cookies [38]. Filipčev et al. [39] also documented betaine reductions spanning from 17 to 28.6% within betaine-fortified wheat-based baked goods. Remarkably high betaine losses (> 90%) were observed after baking of betaine-enriched bread, partly due to consumption of betaine as a nitrogen source by baker’s yeast. In a broader context, betaine stability during food processing varies depending on the type of food and the cooking method used. Although betaine is generally thermostable, losses can occur due to factors such as dissolution in the cooking water, removal during draining, consumption by microorganisms, or release from the food matrix. Our study shows the adverse effects of high temperature and duration of post-processing, which are especially noteworthy in the context of 3D-printing technology. Here, the use of both the AF and VD proved a non-increasing destruction of betaine with increasing infill level. Despite the losses, depending on infill level, one portion (30 g) of air-fried snack could satisfy 69–83% of the recommended dose of 500 mg of betaine per serving [40], while one portion of VD snack could satisfy 80–93% of the recommended dose.

#### 3.2.2. Dimensional Stability of Post-Processed 3D Snacks

The length and width of the snacks deviated by up to 13% after post-processing, while the height stability ranged from 91 to 101% (Table 3). The height stability was only influenced by post-processing technique (*p* = 0.007), while the effect of infill level was insignificant (*p* = 0.153). On the other hand, the post-processing technique interacting with infill level influenced length deviation (*p* = 0.008), but neither had a significant influence on width deviation (*p* = 0.085). In general, the highest dimensional stability with minimal shape deformation was achieved when the snacks were post-processed in DO (Table 3). Similarly, Huang et al. [29] reported that infill density had no effect on the dimensional stability of 3D-printed brown rice paste, while Feng et al. [23] found a strong effect of infill level on the degree of deformation and the quality of the products. They found that when yam-potato snacks were air-fried, the size of the product shrank and deformed to different degrees due moisture removal, although the height of all samples remained similar. This is consistent with our results, since height was the least deformed of all dimensions. In agreement with our results, Feng et al. [23] found that the dimensional properties of 3D-printed snacks were affected by the type of post-processing. They found that the dimensions of hot-dried products were significantly smaller than those of microwave vacuum-dried and freeze-dried products, likely due to greater volume shrinkage caused by moisture evaporation. Theagarajan et al. [41] also confirmed the influence of different post-processing techniques (blanching, steaming, roasting, microwaving, shallow, and deep-frying) on the structural stability of 3D-printed shapes prepared from rice starch. Liu et al. [25] showed that the diameter and height of 3D-printed potato-based snacks decreased during post-processing in an air fryer, but generally, the non-fried and fried samples captured the overall morphological properties of the designed geometry well.

#### 3.2.3. 3D Snack Porosity and Texture

The infill level and the post-processing technique had an interacting effect on the porosity (*p* = 0.036 and *p* < 0.001, respectively), whereby in the Pareto chart the linear term of post-processing technique was dominant (Figure 2). The highest porosity was observed for AF snacks at all three infill levels (Table 3). Different parameters of post-processing techniques, such as heat transfer, temperature, drying time, etc., lead to more or less pronounced formation and expansion of pores inside the snack [42]. For example, the loss of water during baking tends to lift the printed filaments, consequently increasing the distance between layers [43]. In the study by Derossi et al. [42], the overall porosity of baked 3D-printed wheat snacks was 20–30% higher than the designed porosity, which was attributed to the formation of additional pores during dough deposition and baking. In this study, the increase in infill level reduced the porosity of snacks, i.e., porosity was negatively but weakly correlated to infill levels (r = −0.45). Hence, the porosity of snacks with 20% infill level was the highest in all three post-processing techniques (Table 3). This is in agreement with Varghese et al. [44] who showed reducing porosity of oven-baked 3D-printed cookies with increasing infill level. Feng et al. [24] also showed that higher infill levels resulted in a lower porosity and denser structure of yam-potato 3D snacks. Precisely, their porosity values were around 50% and 30% for samples with infill levels of 20% and 50%, respectively. These authors also showed that porosity is affected not only by the infill level but also by the infill pattern.

Textural properties are important for sensory acceptance of snack products [45]. Crunchiness and crispness are the most desired properties for starch-based snack products [27]. “Crispy” is sometimes used to characterize attributes described by others as “crunchy” [46], and some researchers consider the terms interchangeable due to a strong correlation between crispness and crunchiness [47]. Crunchiness is mainly influenced by processing conditions, morphological structures, composition and hydration [48]. In this study, snack crunchiness was influenced by the interaction of post-processing technique and infill level (*p* < 0.001), whereby the Pareto chart shows that the post-processing technique had a dominant effect on crunchiness (Figure 2). In general, higher crunchiness was achieved with a higher infill level for all three post-processing techniques (Table 3). However, with increasing infill level from 20% to 40%, the crunchiness of the AF and VD snacks increased about 2-fold, whereas the crunchiness of the DO-40 snack was only 17% higher than that of DO-20. Contrary to this research, Chen et al. [27] observed lower crunchiness with a higher infill, as the internal structure of the samples became denser, but these authors varied infill levels in the range of 25–100% and used a different material, namely pumpkin powder. While in this study, the snacks post-processed in the VD showed the lowest crunchiness compared with other post-processes, Monteiro et al. [49] succeeded in producing highly porous and crispy sweet potato chips using a microwave vacuum dryer.

The firmness of the 3D snacks in this study was influenced by the interaction of infill level and post-processing technique (*p* < 0.001). Generally, the firmness increased at higher infill levels, but the effect was more pronounced in the AF and DO snacks than in the VD snacks. The Pareto chart shows a positive quadratic effect and negative linear effect of post-processing, and a positive linear effect of the infill level (Figure 2). The observed positive firmness–infill correlation (r = 0.60) was expected since a higher infill level implies more deposited material and a more stable structure, most likely contributing to snack firmness. Likewise, Liu et al. [36] reported that increasing infill percentage increased the hardness of 3D-printed mashed potatoes. Huang et al. [29] also found that the hardness of 3D-printed shapes of brown rice positively correlated with infill density. On the other hand, Feng et al. [23] established decreased hardness of air-fried potato snacks with increasing porosity. Derossi et al. [43] and Feng et al. [23] also found that the hardness of 3D-printed samples decreased as a function of the number of pores, but no significant correlation between firmness and porosity was found here (r = −0.19; *p* = 0.103). Similar to crunchiness, the lowest firmness was found for the VD snacks, followed by the AF and DO snacks. The determined firmness might be related to the moisture content of the snack as a significant negative correlation was found (r = −0.88, *p* = 0.05), but causality should be confirmed as samples with similar moisture content have significantly different firmness (e.g., samples AF-30 and VD-30, among others). Future studies should, therefore, continue to focus on other variables of post-processing techniques. Brittleness was negatively but weakly (r = −0.19, *p* = 0.030) correlated with firmness and was influenced by the interaction of drying technique and infill level (*p* < 0.001). On average, brittleness was highest in the DO snacks and showed a clear decrease in the AF and DO snacks as infill level increased. Previous studies [23,25] showed how various textures can be achieved with the use of air frying to post-process 3D-printed starch-based snacks. Our study confirmed these results, but additionally shows that VD post-processing limits texture variations with increasing infill levels of cereal-based snacks.

### 3.3. Sensory Profile of 3D-Printed Snacks

Preliminary hedonic sensory analysis and ranking tests of the DO snacks were conducted to determine the most acceptable infill level. While there was no statistically significant difference in appearance, texture and overall liking between snacks with different infills (which were ranked as “moderately liked” to “liked very much”), a snack with an infill level 40% was ranked as “most liked” by 50% and as “second liked” by 33% of panelists. In comparison, the lowest sum of ranks (20) was found for snacks printed with an infill level of 40%, while the sum of ranks for snacks with infill levels of 20% and 30% were 30 and 22, respectively. Therefore, a snack with 40% infill level was selected for further study of the effects of post-processing techniques on sensory parameters.

**Figure 3:**
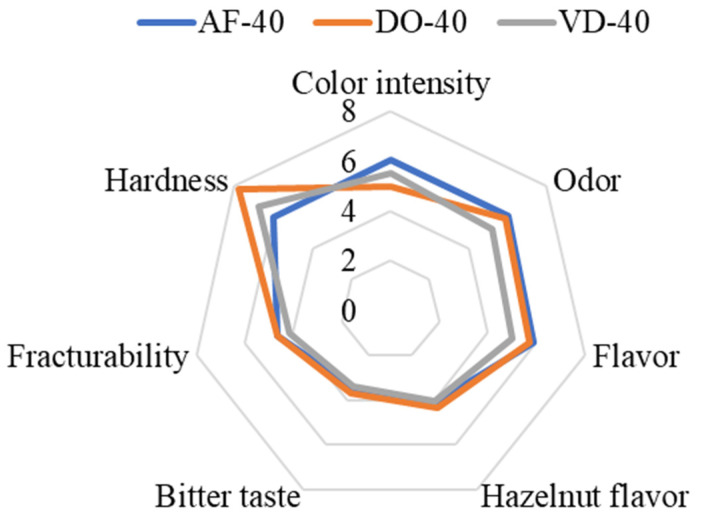
Results of descriptive sensory analysis of snacks printed at 40% infill level, post-processed in an air fryer (AF), drying oven (DO) or vacuum dryer (VD).

The descriptive sensory analysis aimed to identify sensory differences between the snacks post-processed using different techniques. The results showed that there was a significant difference only between the color intensity of the snacks (Figure 3). The DO snacks were significantly lighter compared with the VD and AF snacks (*p* = 0.023; *p* = 0.019, respectively). The observed differences could be due to the magnitude of Maillard reactions occurring at different temperatures [50,51], with Maillard reactions being strongest during air frying. In general, air frying can produce color and flavor similar to deep-fried products, while reducing oil content and the number of polar compounds [27]. Furthermore, the lowest bitterness (3.4) was found in the VD-40 snack, which could also be related to the lack of formation of bitter compounds that normally occur in Maillard reactions [51]. Moreover, the DO-40 snack was significantly harder than the VD-40 snack (*p* = 0.001), but not AF-40.

In the ranking test, AF-40 was the first choice for 56% of the panelists and achieved the highest hedonic scores for texture (7.44). In terms of flavor, the DO-40 snack was the most liked (7.13), while in terms of overall experience, VD-40 was rated highest (6.75). However, all snacks were rated as either “moderately liked” or “liked very much”, with no significant difference in acceptability established.

## 4. Conclusions

While it is well known that 3D-printing can be successfully used to produce snack products with different designs, structures and textures, the choice of post-processing method plays a crucial role in the final physical and sensory properties, as well as the product’s nutritional quality. This study investigated the effects of less explored techniques, specifically air frying and vacuum drying, compared with the commonly used drying oven technique, on the quality of 3D-printed snacks enriched with betaine as a functional ingredient.

Of the three post-processing techniques, betaine was best preserved when the 3D snacks were vacuum-dried, suggesting that a lower post-processing temperature helps to preserve betaine. If higher post-processing temperatures are to be used, a shorter processing time is required to retain the betaine, as was the case with air frying. However, the betaine content, as well as snack texture, is influenced by the interaction of post-processing technique and product design. In general, higher infill levels result in higher snack firmness and crunchiness. Of the post-processes applied, air frying led to the highest crunchiness and porosity and the lowest brittleness of the snacks. The overall influence of post-processing methods on sensory attributes and hedonic preference of cereal-based snacks was shown to be relatively modest. The results show the potential of air frying as a post-processing technique for 3D cereal-based snacks, a technique that can be easily used at home. Vacuum drying also has potential in the preservation of bioactive compounds and obtaining a softer texture, but should be modified to obtain sensory-appealing and high-quality 3D-printed snacks. Further studies should explore a broader application of these techniques to other types of products, taking into account other processing parameters besides temperature.

## Figures and Tables

**Table 1:** Experimental plan and processing conditions.

Snack Label (Technique—Infill Level)	Infill Level (%)	Post-Processing Technique	Post-Processing Duration (min)
AF-20	20	Air frying	5
DO-20	20	Oven drying	20
VD-20	20	Vacuum drying	60
AF-30	30	Air frying	7
DO-30	30	Oven drying	25
VD-30	30	Vacuum drying	90
AF-40	40	Air frying	7
DO-40	40	Oven drying	30
VD-40	40	Vacuum drying	90

**Table 2:** CAD models and 3D-printed dough images and dimensional parameters.

	20% Infill	30% Infill	40% Infill
		**CAD Models**	
Images	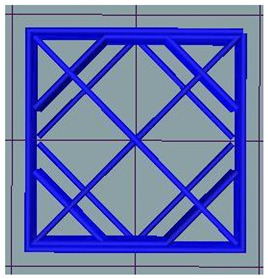	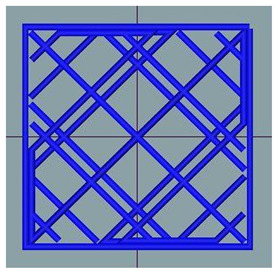	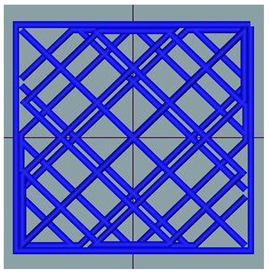
Width/Length/Height (cm)	1.70/1.70/0.70		
	**Printed Dough Shapes**		
Images	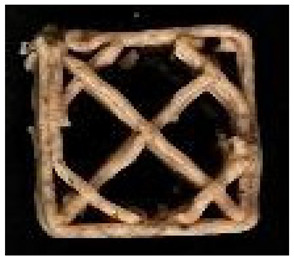	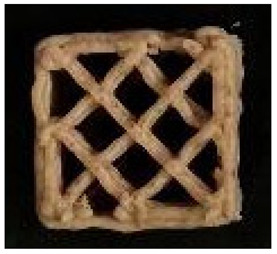	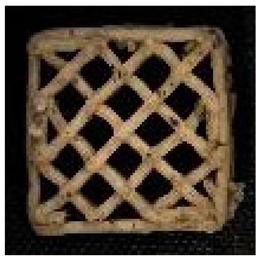
Weight (g)	0.92 ± 0.09 a	0.97 ± 0.09 a	1.21 ± 0.09 b
Width/Length/Height (cm)	1.67 ± 0.03 a/1.69 ± 0.02 a/ 0.61 ± 0.06 a	1.82 ± 0.05 b/1.82 ± 0.05 b/ 0.64 ± 0.03 a	1.82 ± 0.04 b/1.82 ± 0.06 b/ 0.62 ± 0.06 a
Width deviation (%)	−2.33 ± 0.93 a	6.95 ± 2.78 b	7.24 ± 2.47 b
Length deviation (%)	−1.05 ± 0.69 a	6.95 ± 3.06 b	6.76 ± 3.29 b
Height stability (%)	86.86 ± 9.20 a	91.45 ± 4.16 a	89.05 ± 8.00 a

Statistically significant (*p* < 0.05) differences within same raw are marked with different letters.

**Table 3:** Physico-chemical parameters of post-processed 3D-printed snacks.

Snack Label	AF-20	AF-30	AF-40	DO-20	DO-30	DO-40	VD-20	VD-30	VD-40
**Moisture content (%)**	3.83 ± 0.03 b A	3.76 ± 0.04 b B	3.18 ± 0.21 a B	3.89 ± 0.04 c A	2.86 ± 0.02 b A	2.15 ± 0.08 a A	4.49 ± 0.08 b B	3.71 ± 0.03 a B	3.82 ± 0.01 a C
**Betaine** **(mg/100 g)** **(Betaine loss)**	1112.38 ± 12.21 a A (40.19%)	1265.55 ± 10.70 b A (31.95%)	1338.90 ± 5.04 c A (28.01%)	1218.41 ± 17.33 c B (34.49%)	981.61 ± 4.15 b B (47.23%)	845.03 ± 4.01 a B (54.57%)	1280.84 ± 6.41 a B (31.14%)	1340.26 ± 10.28 b C (27.94%)	1497.39 ± 3.87 c C (19.50%)
Height stability (%) *	93.31 ± 5.38 a A	92.38 ± 5.46 a A	93.31 ± 5.38 a A	96.85 ± 3.84 a A	101.66 ± 2.61 a B	99.42 ± 1.48 a A	91.08 ± 4.76 a A	93.22 ± 7.01 a A	99.92 ± 4.65 a A
Width deformation (%) *	10.96 ± 2.25 a B	10.91 ± 1.69 a A	10.96 ± 2.25 a A	7.78 ± 2.52 a A	10.62 ± 1.18 a A	11.93 ±1.48 a A	10.86 ± 1.92 a B	11.84 ± 1.32 a A	12.78 ± 1.33 a A
Length deformation (%) *	11.51 ± 1.32 a A	9.47 ± 1.90 a A	11.51 ± 1.32 a A	8.30 ± 2.41 a A	10.62 ± 1.10 a A	9.95 ± 1.20 a A	12.61 ± 1.96 a A	9.35 ± 1.64 a A	13.19 ± 1.06 a A
**Porosity (%)**	44.14 ± 1.06 b C	43.66 ± 3.83 ab B	41.23 ± 2.91 a B	37.58 ± 2.06 b A	33.42 ± 2.91 a A	32.30 ± 2.46 a A	40.37 ± 4.44 c B	34.05 ± 2.28 b AB	30.28 ± 0.27 a A
**Firmness (N)**	27.12 ± 4.49 a A	40.62 ±8.48 b B	47.57 ± 10.09 bc B	33.47 ± 4.70 a B	45.07 ± 2.31 b B	58.32 ± 5.53 c B	17.07 ± 2.35 a C	18.36 ± 1.65 a A	26.73 ± 2.85 b A
**Crunchiness (Nmm)**	22.26 ± 1.82 a B	47.53 ± 18.47 ab C	54.12 ± 17.90 b C	29.66 ± 3.43 a C	31.34 ± 4.36 a B	34.63 ± 4.86 a B	8.37 ± 1.23 a A	10.67 ± 1.17 a A	19.16 ± 2.88 b A
**Brittleness**	2.78 ± 0.71 a A	2.55 ± 0.51 a A	2.43 ± 0.21 a A	3.42 ±b0.20 b B	3.08 ± 0.22 b B	2.38 ± 0.56 a A	2.87 ± 0.27 b AB	2.47 ± 0.23 a A	2.63 ± 0.15 b A

* Statistically significant differences within same post-processing technique are marked with lowercase letters, while statistically significant differences within same infill levels are marked with uppercase letters.

## Data Availability

Data are contained within the article.
